# Supplementary material for: Lrig1-expression confers suppressive function to CD4+ cells and is essential for averting autoimmunity via the Smad2/3/Foxp3 axis
Source: Nat Commun. 2023 Sep 4;14:5382. doi: 10.1038/s41467-023-40986-4 (PMC10477202; doi:10.1038/s41467-023-40986-4)

Supplementary Information

# Lrig1-expression confers suppressive function to CD4$^{+}$ cells and is essential for averting autoimmunity via the Smad2/3/Foxp3 axis

**Authors:** Jae-Seung Moon[#1,8], Chun-Chang Ho[#1,2], Jong-Hyun Park[3], Kyungsoo Park[4], Bo-Young Shin[1,2], Su-Hyeon Lee[1], Ines Sequeira[5], Chin Hee Mun[6], Jin-Su Shin[1,2], Jung-Ho Kim[2], Beom Seok Kim[2], Jin-Wook Noh[2], Eui-Seon Lee[2], Ji Young Son[2], Yuna Kim[1], Yeji lee[2], Hee Cho[1], SunHyeon So[2], Jiyoon Park[1], Eunsu Choi[2], Jong-Won Oh[1], Sang-Won Lee[6], Tomohiro Morio[7], Fiona M. Watt[5], Rho Hyun Seong[4], Sang-Kyou Lee[1,2,*]

**Affiliations:**

[1]Department of Biotechnology, Yonsei University College of Life Science and Biotechnology, Seoul, Republic of Korea

[2]Good T cells, Inc., Seoul, Republic of Korea

[3]Convergence Research Center for Diagnosis, Treatment and Care System of Dementia, Korea Institute of Science and Technology, Seoul, Republic of Korea

[4]Department of Biological Sciences and Institute of Molecular Biology and Genetics, Seoul National University, Seoul, Republic of Korea

[5]Centre for Stem Cells and Regenerative Medicine, King's College London, Guy's Hospital, London, UK

[6]Division of Rheumatology, Department of Internal Medicine, Yonsei University College of Medicine, Seoul, Republic of Korea

[7]Department of Pediatrics and Developmental Biology, Graduate School of Medical and Dental Sciences, Tokyo Medical and Dental University (TMDU), Tokyo, Japan

[8]Present address: Division of Immunology and Rheumatology, Department of Medicine, Stanford University School of Medicine, Stanford, CA, USA

[#]These authors equally contributed.

*Email: sjrlee@goodtcells.co.kr

## Supplementary Figures

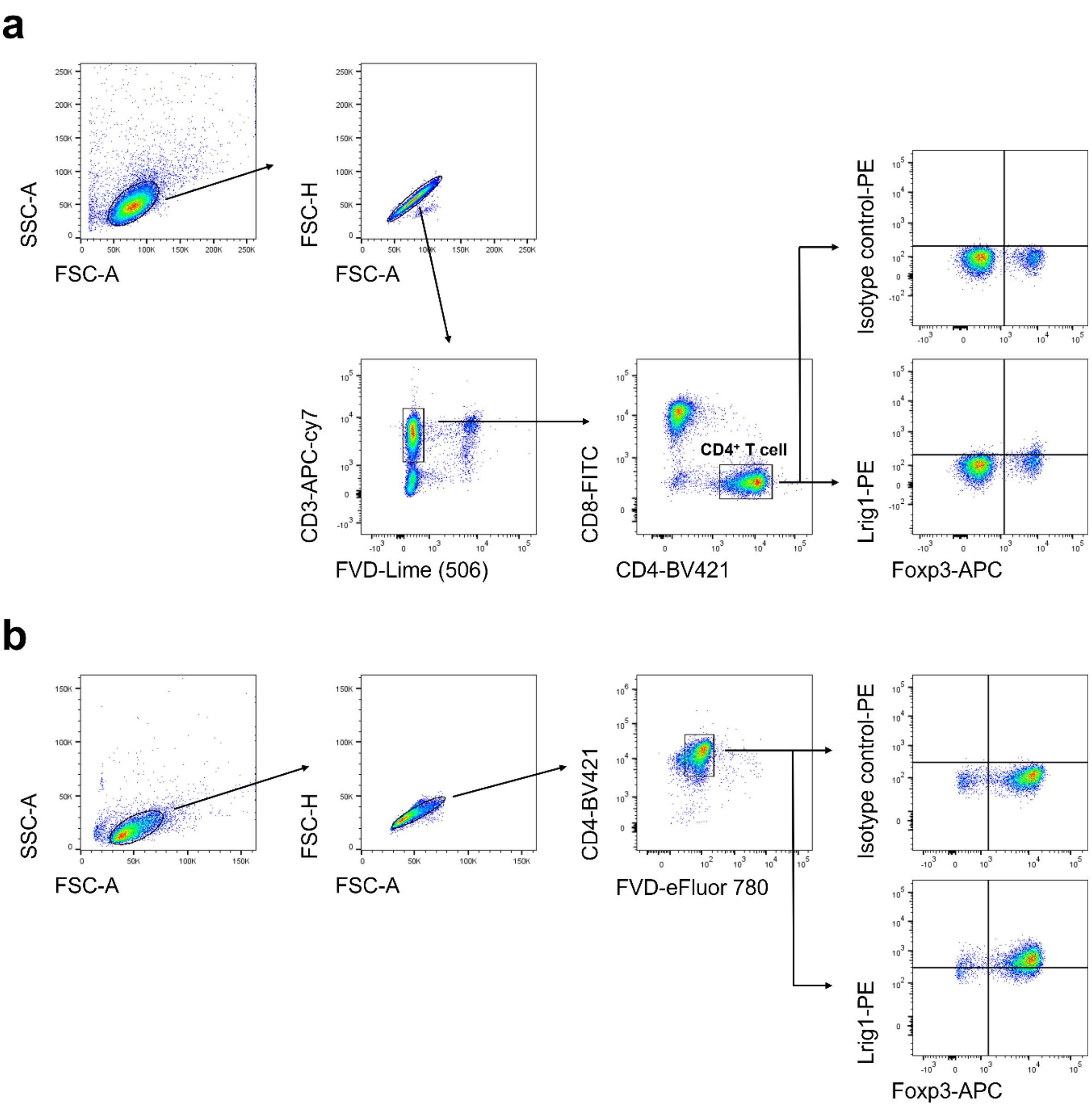


**Supplementary Fig. 1: Gating strategy used for FACS analysis. a** Gating strategy to analyze lymphocytes from spleen, lymph node, thymus, or spinal cord. The population of Lrig1$^+$Foxp3$^+$ cells were analyzed in CD4$^+$ T cells (presented on Fig. 1e, Fig. 6c). **b** Gating strategy to analyze *in vitro* differentiated iT$_{reg}$ cells. iT$_{reg}$ cells were differentiated from isolated CD4$^+$ naïve T cells. The population of Lrig1$^+$ cells or Foxp3$^+$ cells were analyzed (presented on Fig. 1b-d, Fig. 5a, c).

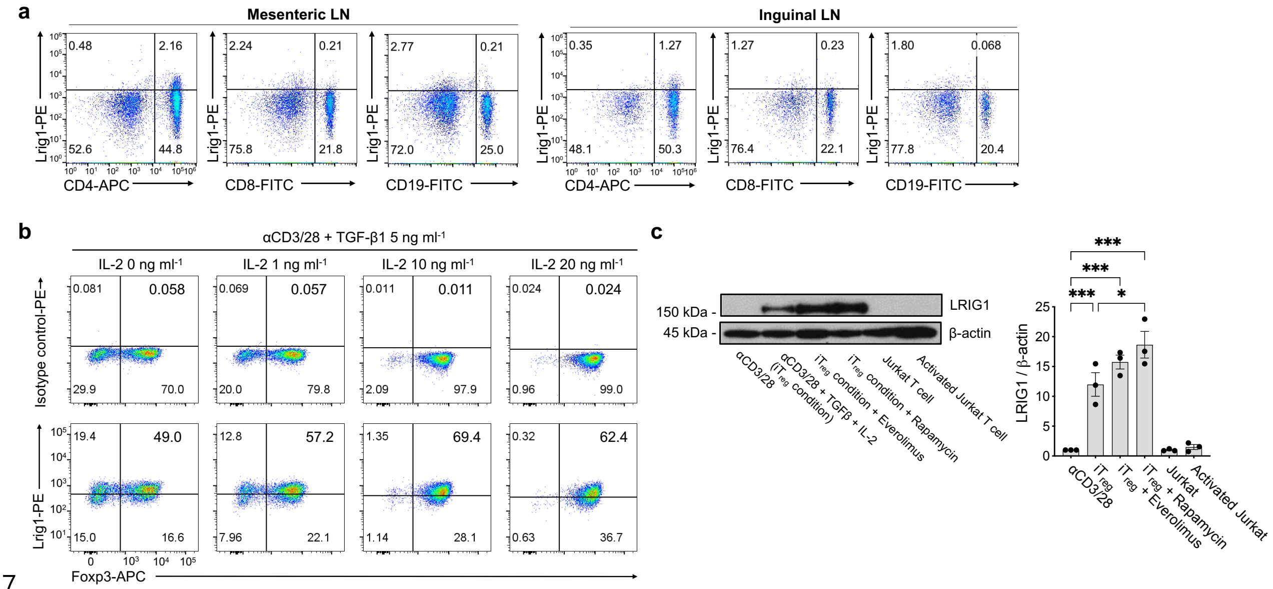


**Supplementary Fig. 2: The Lrig1 expressing cells among CD4+, CD8+ or CD19+ mouse immune cells or human T cells.** **a** The level of Lrig1+ cells in immune cells (CD4+, CD8+ T cells, or CD19+ B cells) from mesenteric or inguinal lymph node in the mouse. **b** The change of Lrig1 expression on CD4+Foxp3+ T cells differentiated from naïve T cells with different IL-2 concentrations compared to isotype control. **c** The level of human LRIG1 protein in activated human T cells, human iT$_{reg}$ cells, human iT$_{reg}$ cells cultured with either Everolimus or Rapamycin, Jurkat T cells, or activated Jurkat T cells was examined by western blot using anti-human LRIG1 antibody, and β-actin was used as the protein amount control. Relative expression of LRIG1 based on β-actin expression was quantified by Image J software (n=3). Data are expressed as mean ± S.E.M (*P = 0.03, ***P < 0.001). Ordinary one-way ANOVA with Tukey's multiple comparisons test (**c**). Source data are provided as a Source Data file and at the end of Supplementary Information.

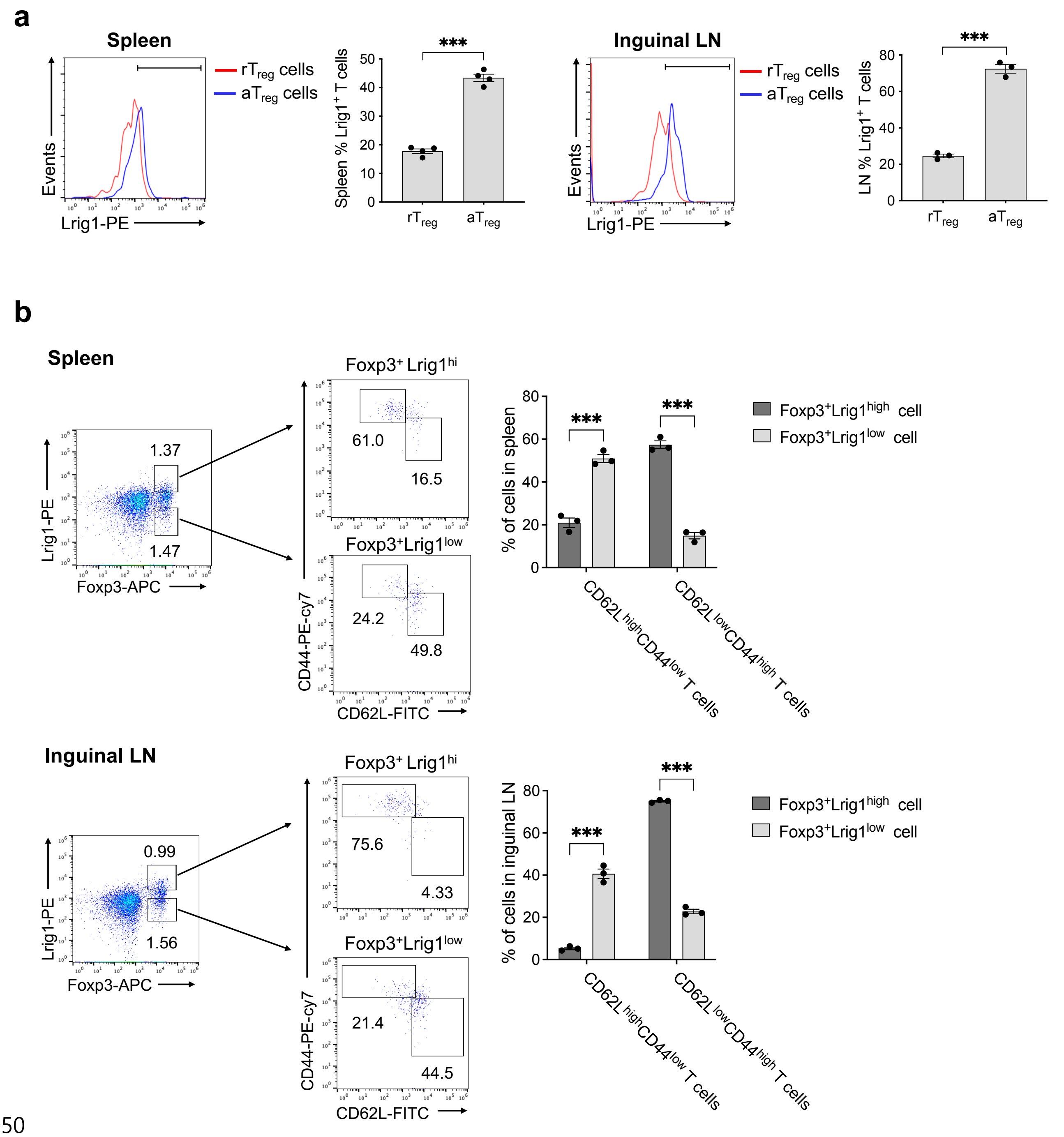


**Supplementary Fig. 3: Higher expression of Lrig1 is detected on the surface of activated $T_{reg}$ cells ($aT_{reg}$) than resting $T_{reg}$ cells ($rT_{reg}$). a** The level of Lrig1 expressing cells among $aT_{reg}$ ($CD62L^{-}CD44^{+}$), or $rT_{reg}$ ($CD62L^{+}CD44^{-}$) cells in $CD4^{+}Foxp3^{+}$ T cells purified from spleen (n=4) or inguinal LN (n=3) of Foxp3-IRES-GFP mice. Data are expressed as mean ± S.E.M (***P < 0.0001). **b** $CD4^{+}$ T cells from the spleen or inguinal LN were separated into $Foxp3^{+}Lrig1^{hi}$ or $Foxp3^{+}Lrig1^{low}$ T cells, and the level of CD44 and CD62L expression

was analyzed (n=3 for each group). Data are expressed as mean ± S.E.M (***P < 0.0001). Data are representative of at least three independent experiments. Statistical significance is determined by two-tailed unpaired t-test (**a**) or two-way ANOVA with Šídák's multiple comparisons test (**b**). Source data are provided as a Source Data file.

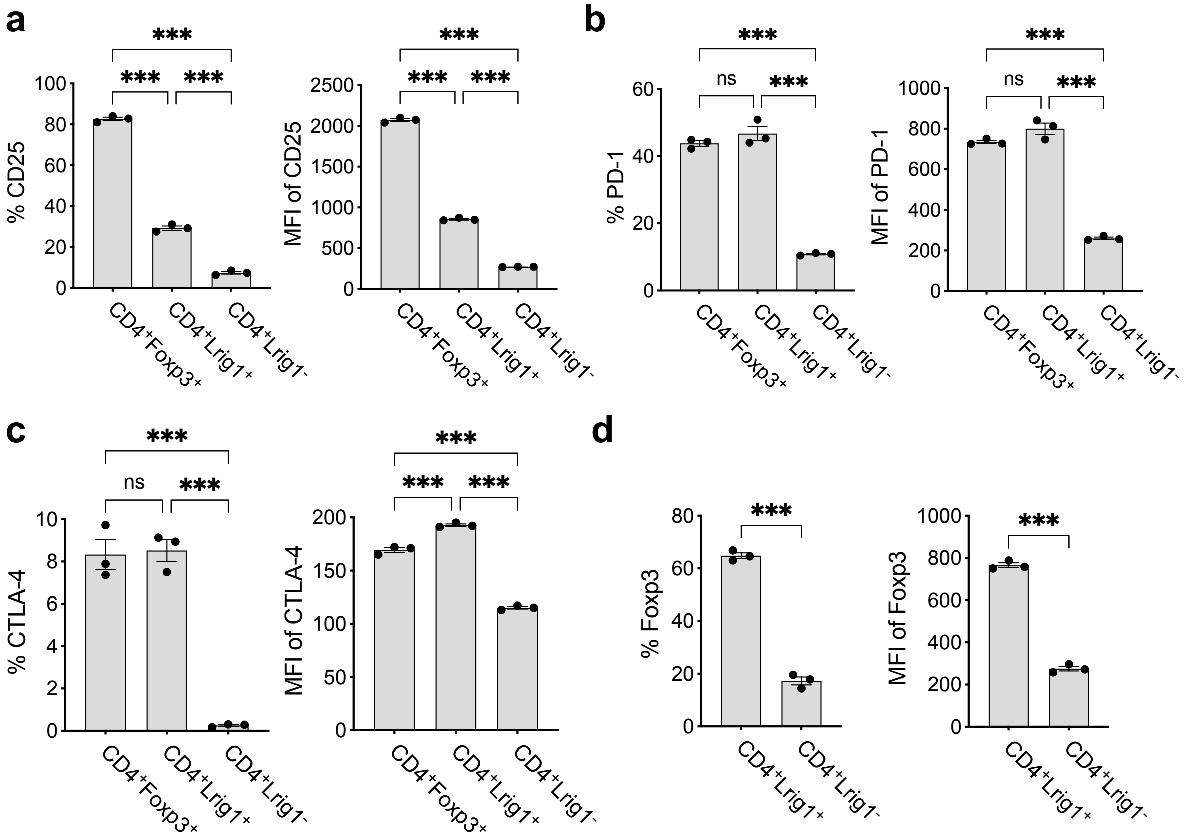


**Supplementary Fig. 4: CD4$^+$Lrig1$^+$ T cells highly express suppressive markers to regulate effector T cell proliferation compared to CD4$^+$Lrig1$^-$ T cells. a-d** The percentage (*left*) or MFI (*right*) of the expression level of CD25, PD-1, CTLA-4, or Foxp3 in CD4$^+$Foxp3$^+$, CD4$^+$Lrig1$^+$, or CD4$^+$Lrig1$^-$ T cells during the suppression assay co-cultured with effector CD4$^+$ T cells (n=3 for each group). Data are expressed as mean ± S.E.M (in **a-d**, ***P < 0.001). Ordinary one-way ANOVA with Tukey's multiple comparisons test (**a-c**) and two-tailed unpaired t-test (**d**). Source data are provided as a Source Data file.

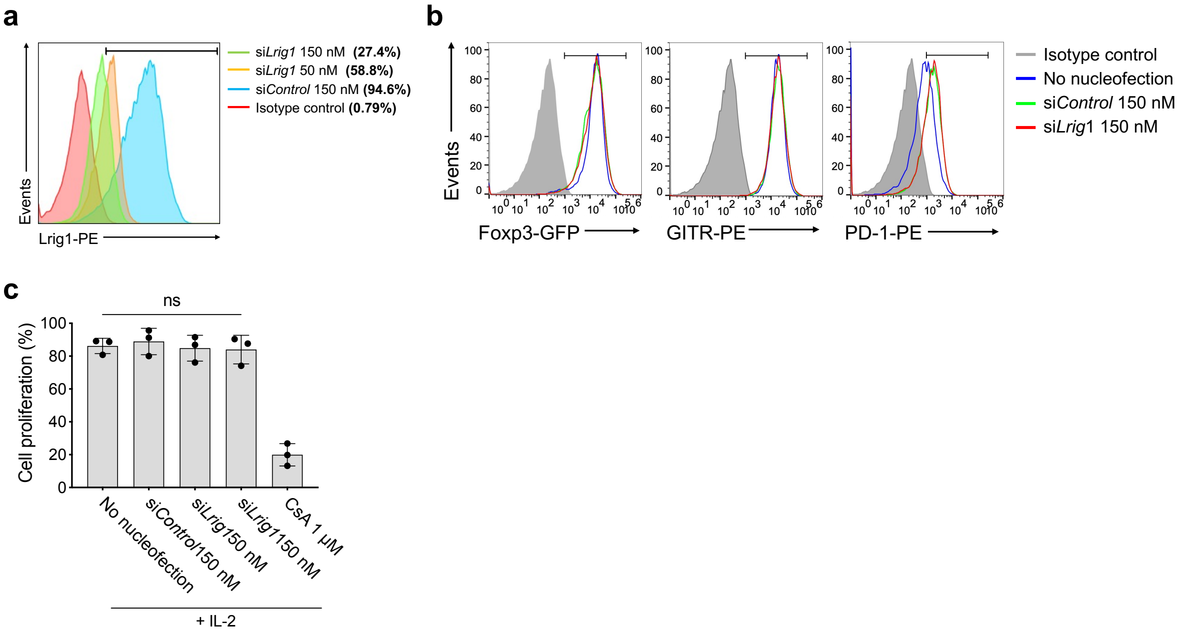


**Supplementary Fig. 5: The reduced expression of Lrig1 on $T_{reg}$ cells does not influence the expression of other $T_{reg}$ markers and $T_{reg}$ cell proliferation. a** Representative histogram showing the level of Lrig1 expression on the cell surface of $iT_{reg}$ cells knocked down by 50 nM or 150 nM of siRNA targeting *Lrig1* (si*Lrig1*) or 150 nM of scrambled sequences (si*Control*). **b** Expression of Foxp3, GITR, or PD-1 on si*Lrig1*- or si*Control*-nucleofected $iT_{reg}$ cells. **c** siRNA-nucleofected naïve $CD4^+$ T cells were labeled with cell proliferation eFlour670 dye and induced to differentiate into $iT_{reg}$ cells (n=3). $iT_{reg}$ cells were incubated with IL-2 for another 48 h to measure the level of $iT_{reg}$ cell proliferation. Cyclosporin A (CsA) was used as a positive control. Data are expressed as mean ± S.E.M. ns, not significant. Ordinary one-way ANOVA with Tukey's multiple comparisons test (**c**). Data are representative of three independent experiments. Source data are provided as a Source Data file.

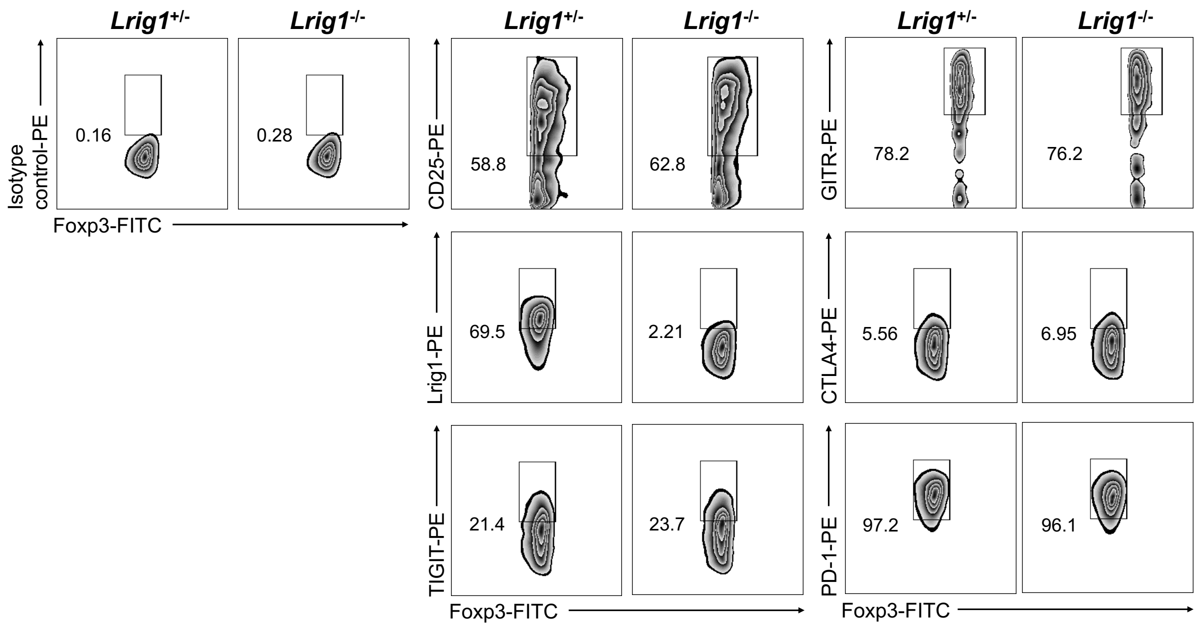


**Supplementary Fig. 6: The expression of the suppressive markers on $T_{reg}$ cells is not altered in *Lrig1*-deficient mice.** The level of CD25, GITR, Lrig1, CTLA-4, TIGIT, or PD-1 in Foxp3$^+$ i$T_{reg}$ cells differentiated from naïve CD4$^+$ T cells in *Lrig1*$^{+/-}$ or *Lrig1*$^{-/-}$ mice. Data are representative of three independent experiments.

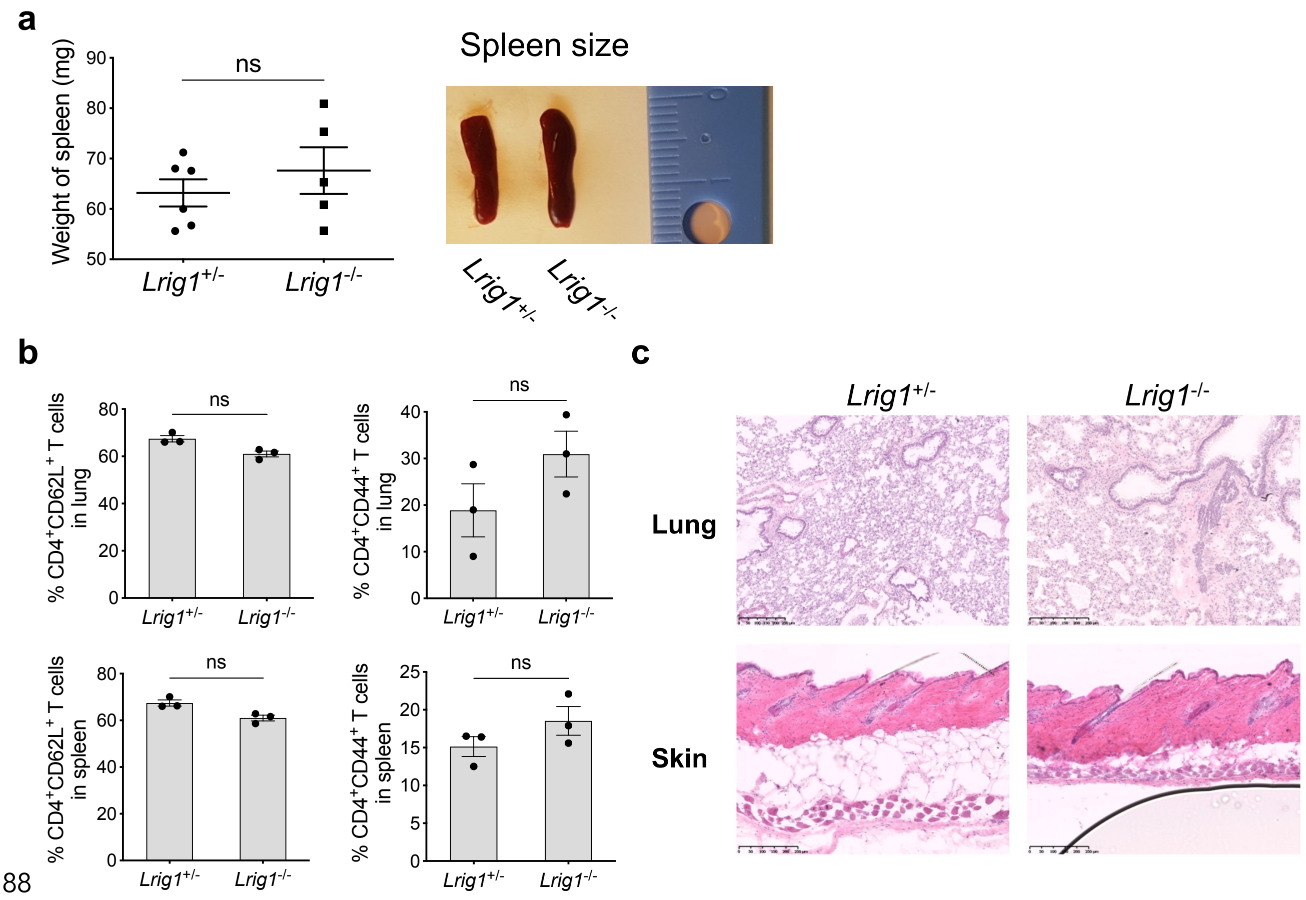


**Supplementary Fig. 7: Abnormal T cell subset composition changes and excessive inflammation are not observed in *Lrig1* null mice. a** Body weight and the size of the spleen from *Lrig1*$^{+/-}$ or *Lrig1*$^{-/-}$ mice were compared (*Lrig1*$^{+/-}$ mice; n=6, *Lrig1*$^{-/-}$ mice; n=5). **b** Lung-resident or splenic lymphocytes were prepared from *Lrig1*$^{+/-}$ or *Lrig1*$^{-/-}$ mice (n=3 for each group), and the level of CD4$^{+}$CD62L$^{+}$ or CD4$^{+}$CD44$^{+}$ T cells was examined. **c** Representative images of hematoxylin and eosin (H&E) staining in lung and skin tissue from *Lrig1*$^{+/-}$ or *Lrig1*$^{-/-}$ mice. The scale bar indicates 200 μm. Error bars represent the mean ± S.E.M. ns not statistically significant. Statistical significance was determined by two-tailed unpaired student's t-test (**a, b**). Three independent experiments were merged. Source data are provided as a Source Data file.

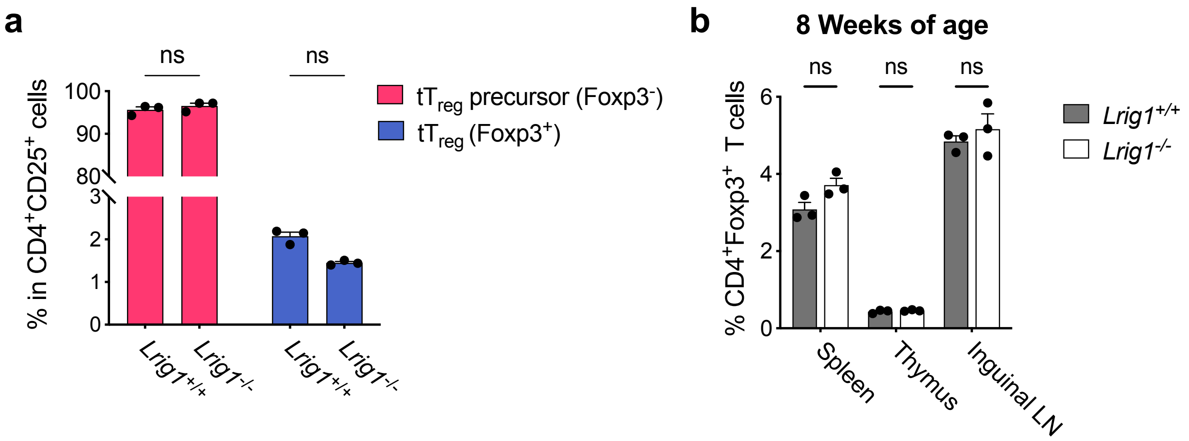


**Supplementary Fig. 8: Lrig1 deficiency did not alter $tT_{reg}$ development in the thymus and the proportion of $CD4^+Foxp3^+$ T cells in the spleen, thymus, and lymph node. a** Proportion of thymic $T_{reg}$ ($tT_{reg}$) precursor ($CD4^+CD25^+Foxp3^-$ cells) and $tT_{reg}$ ($CD4^+CD25^+Foxp3^+$ cells) cells in thymic lymphocytes from *Lrig1*$^{+/+}$ or *Lrig1*$^{-/-}$ mouse (n=3). **b** Proportion of $CD4^+Foxp3^+$ T cells in spleen, thymus, or inguinal LN form *Lrig1*$^{+/+}$ or *Lrig1*$^{-/-}$ mouse (n=3). Error bars represent the mean ± S.E.M. ns not significant. Statistical significance was determined by two-way ANOVA with Tukey's (**a**) or two-way ANOVA with Šídák's (**b**) multiple comparisons test. Source data are provided as a Source Data file.

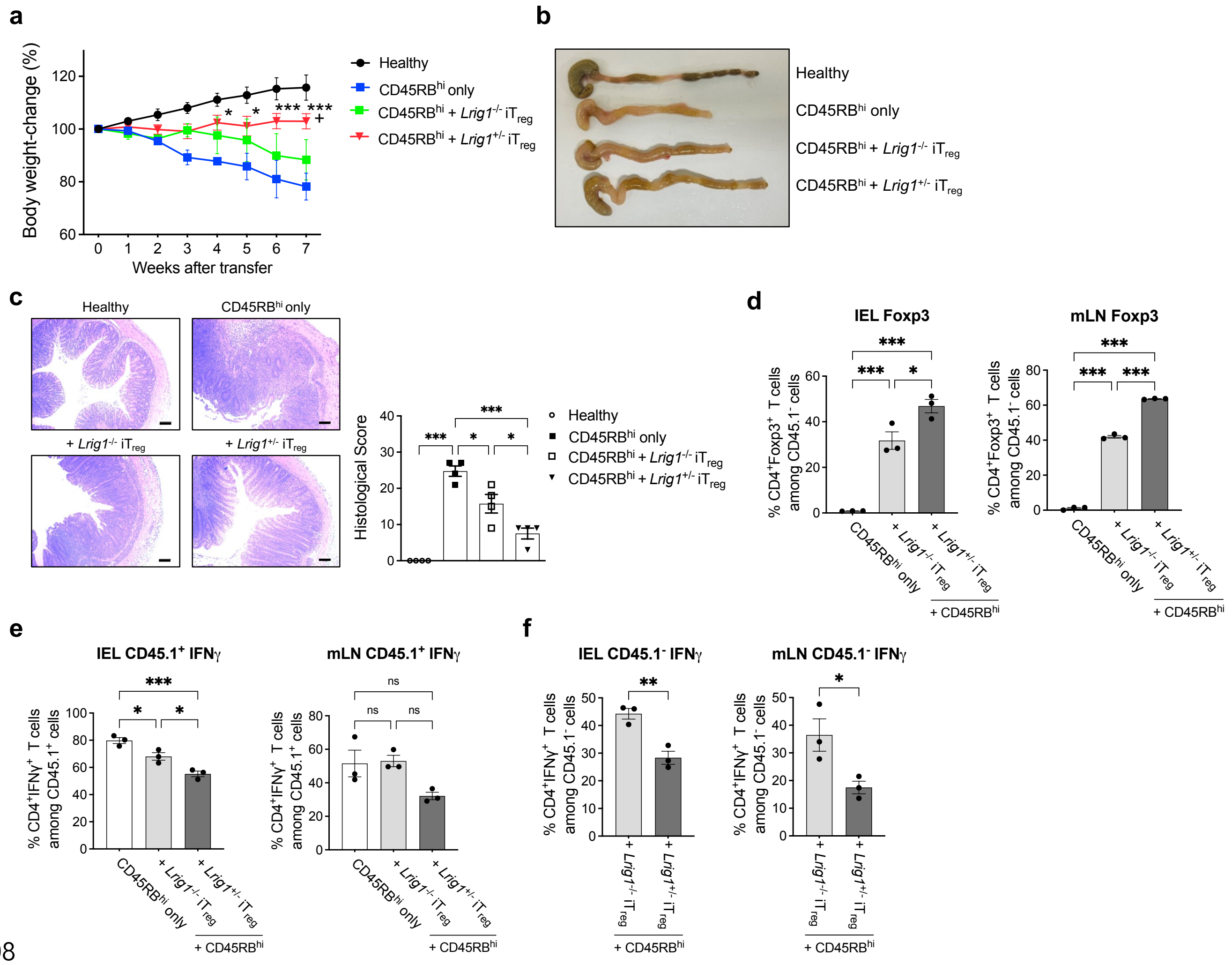


**Supplementary Fig. 9: *Lrig1*-expressing iT$_{reg}$ cells show a significant suppressive function *in vivo* by alleviating the inflammatory responses in IBD mice compared to *Lrig1*-deficient iT$_{reg}$ cells. a** Body weight change of normal C56BL/6 (n=4) or *Rag1*-knockout mice transferred with CD45.1$^{+}$CD4$^{+}$CD45RB$^{high}$ T cell only (n=4), or together with iT$_{reg}$ cells from *Lrig1*$^{+/-}$ (n=4) or *Lrig1*$^{-/-}$ (n=4) mice. Data are expressed as mean ± S.E.M (week 4 *P = 0.0454; week 5 *P = 0.0324; week 6 ***P = 0.0008; week 7 +P = 0.0463, ***P = 0.0001). * symbol shows versus CD45RB$^{hi}$ only group and + symbol shows versus CD45RB$^{hi}$ + *Lrig1*$^{-/-}$ iT$_{reg}$ cells. **b** Representative image of macroscopic changes in the colon from each recipient group. **c** Representative images of hematoxylin and eosin (H&E) staining (*left*) and the combined histopathological clinical scores (*right*) of large intestines (n=4). Scale bar indicates 10 µm. Data are expressed as mean ± S.E.M (*P = 0.01 CD45RB$^{hi}$ only vs + *Lrig1*$^{-/-}$ iT$_{reg}$, *P = 0.02 + *Lrig1*$^{-/-}$ iT$_{reg}$ vs + *Lrig1*$^{+/-}$ iT$_{reg}$, ***P < 0.001). **d** The level of CD4$^{+}$Foxp3$^{+}$ cells among CD45.1$^{-}$ cells in the re-stimulated lymphocytes from the colonic lamina propria (intraepithelial lymphocytes, IEL), or mesenteric lymph node (mLN) of each recipient group (n=3). Data are

expressed as mean ± S.E.M (*P = 0.02, ***P < 0.001). **e, f** Quantification of $CD4^+IFN\gamma^+$ T cells in IEL or mLN from $CD45.1^+$ (**e**) (n=3) or $CD45.1^-$ (**f**) (n=3) cells. Data are expressed as mean ± S.E.M (in **e**, *P = 0.03 $CD45RB^{hi}$ only vs + *Lrig1*$^{-/-}$ $iT_{reg}$, *P = 0.02 + *Lrig1*$^{-/-}$ $iT_{reg}$ vs + *Lrig1*$^{+/-}$ $iT_{reg}$, ***P < 0.001) (in **f**, *P = 0.04, **P = 0.006). ns, not significant. Statistical significance was determined by two-way ANOVA with Tukey's multiple comparisons test (**a**), one-way ANOVA with Tukey's multiple comparisons test (**c-e**), and two-tailed unpaired t-test (**f**). Source data are provided as a Source Data file.

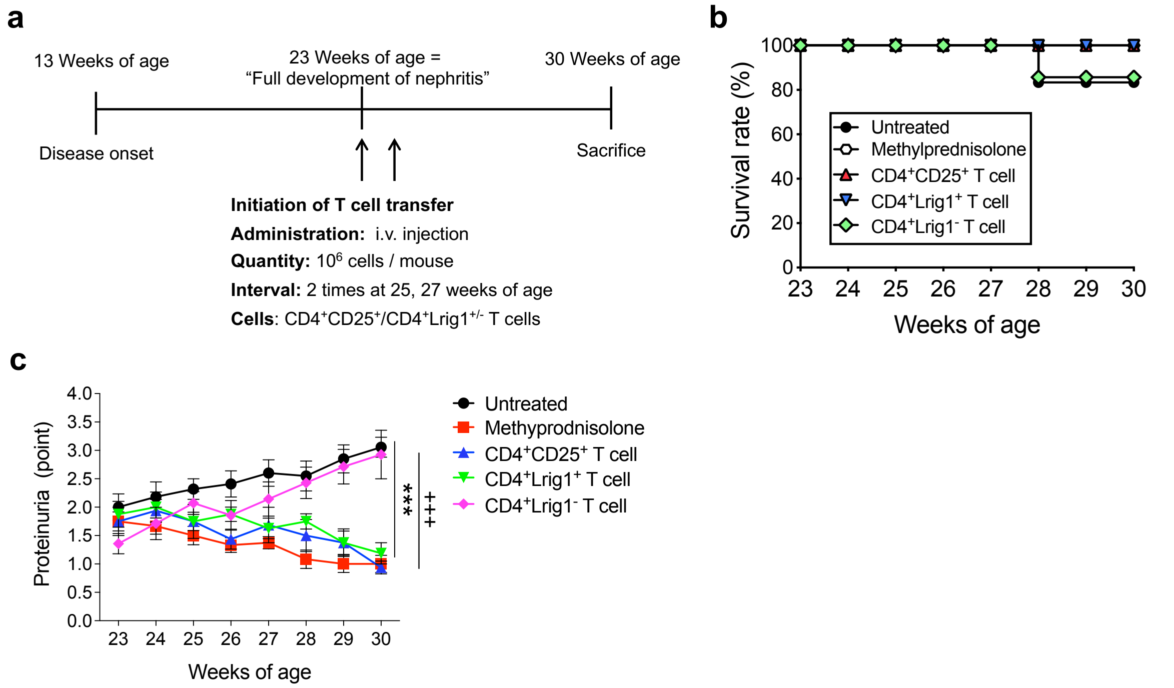


**Supplementary Fig. 10: Adoptive transfer of CD4$^+$Lrig1$^+$ T cells into lupus-prone mice alleviates the autoimmune lupus phenotypes. a** Scheme of the treatment with PBS (Untreated) (n=11) or methylprednisolone (140 µg/mouse) (n=12), of adoptive transfer of CD4$^+$CD25$^+$ T cells (10$^6$ cells/mouse) (n=8), CD4$^+$Lrig1$^+$ T cells (10$^6$ cells/mouse) (n=8) or CD4$^+$Lrig1$^-$ T cells (10$^6$ cells/mouse) (n=7) into (NZB/NZW) F1 mice. **b** Survival rate of the lupus-prone mice with different treatments. **c** The level of proteinuria in each treatment group was detected using a urine dipstick once a week. The scale of proteinuria was based on a scale of 0 (none or trace) to 4 (>1000 mg/dl). Data are expressed as mean ± S.E.M (***P < 0.0001, +++P < 0.0001). In (**c**), + symbol shows versus CD4$^+$Lrig1$^-$ T cells-injected mice and * symbol shows versus untreated mice. Statistical significance was determined by two-way ANOVA with Tukey’s multiple comparisons test at 30 weeks of age. Source data are provided as a Source Data file.

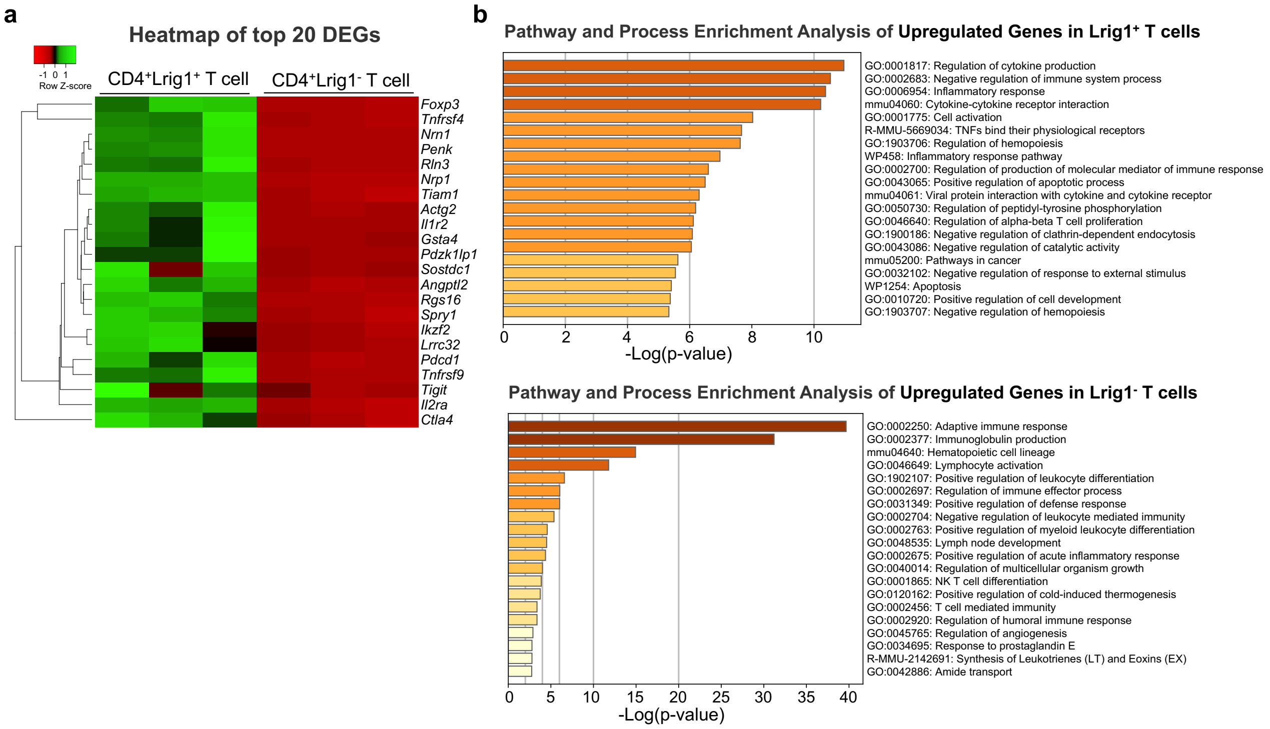


**Supplementary Fig. 11: Pathway enrichment analysis of the top 20 differentially expressed genes (DEGs) in CD4$^+$Lrig1$^+$ T cells. a** Heatmap representing the top 20 DEGs in CD4$^+$Lrig1$^+$ T cells compared with CD4$^+$Lrig1$^-$ T cells. **b** Pathway and process enrichment analysis, including GO Biological Processes, KEGG Pathway, Reactome Gene Sets, CORUM and WikiPathways performed with Metascape using upregulated genes in Lrig1$^+$ or Lrig1$^-$ T cells.

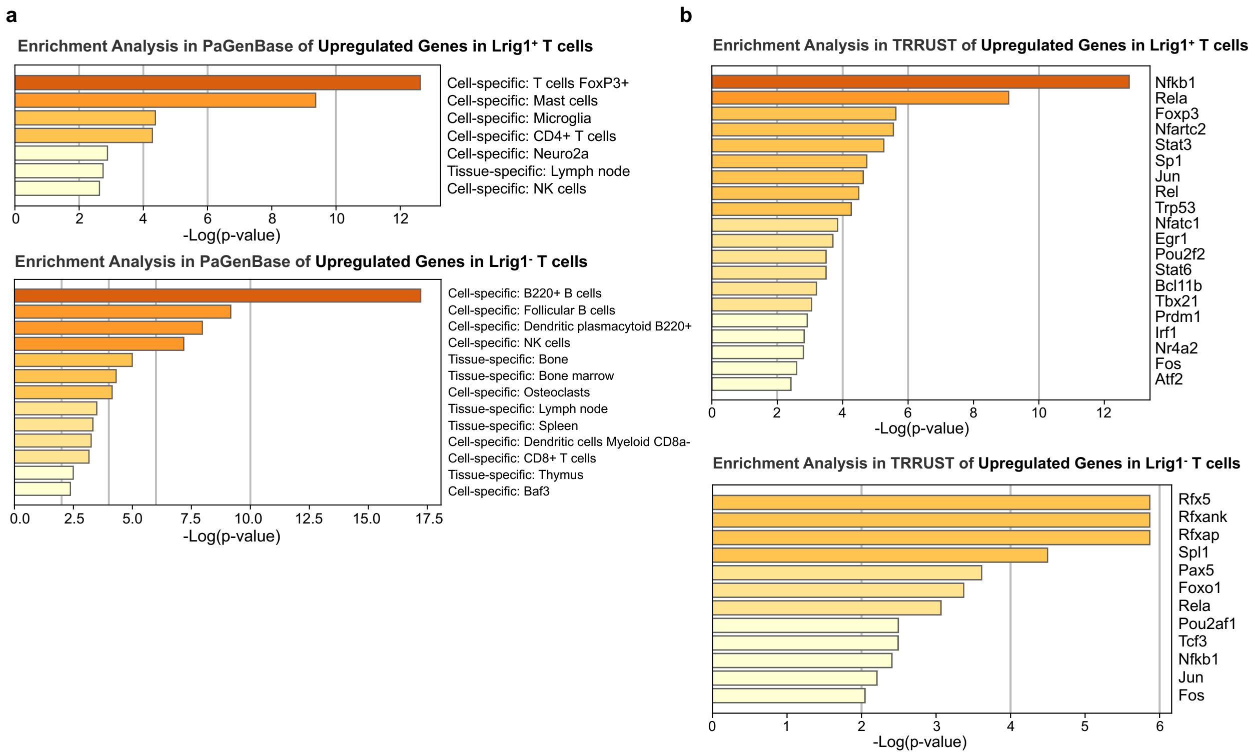


**Supplementary Fig. 12: Enrichment analysis of upregulated genes in Lrig1$^+$ or Lrig1$^-$ T cells using PaGenBase or TRRUST. a** Enrichment analysis showing the association of specific cell type with the upregulated Lrig1$^+$ (*upper*) or Lrig1$^-$ (*lower*) T cells performed with PaGenBase analysis in Metascape. **b** Enrichment analysis representing the regulatory genes to express the upregulated genes in Lrig1$^+$ (*upper*) or Lrig1$^-$ (*lower*) T cells performed with TRRUST analysis in Metascape.

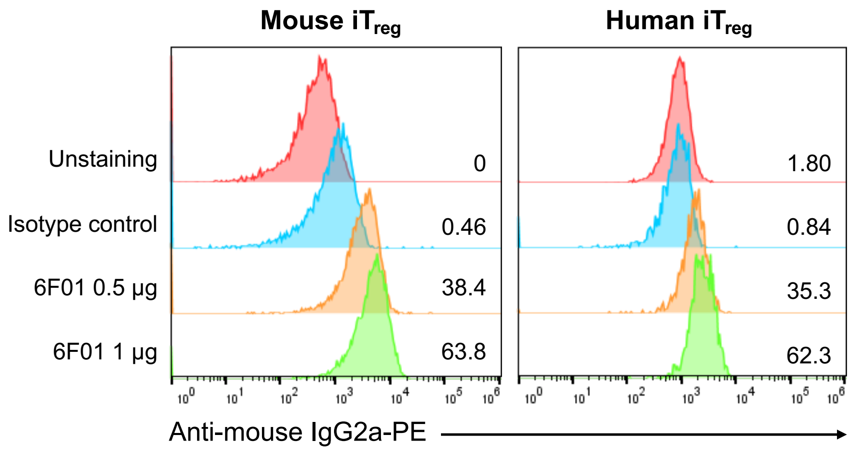


**Supplementary Fig. 13: Surface staining of mouse and human iT$_{reg}$ cells with 6F01 mAb.** Mouse and human iT$_{reg}$ cells differentiated from naïve CD4$^+$ T cells were stained with two different concentrations of 6F01 with isotype-matched IgG as a control. Data are representative of three independent experiments.

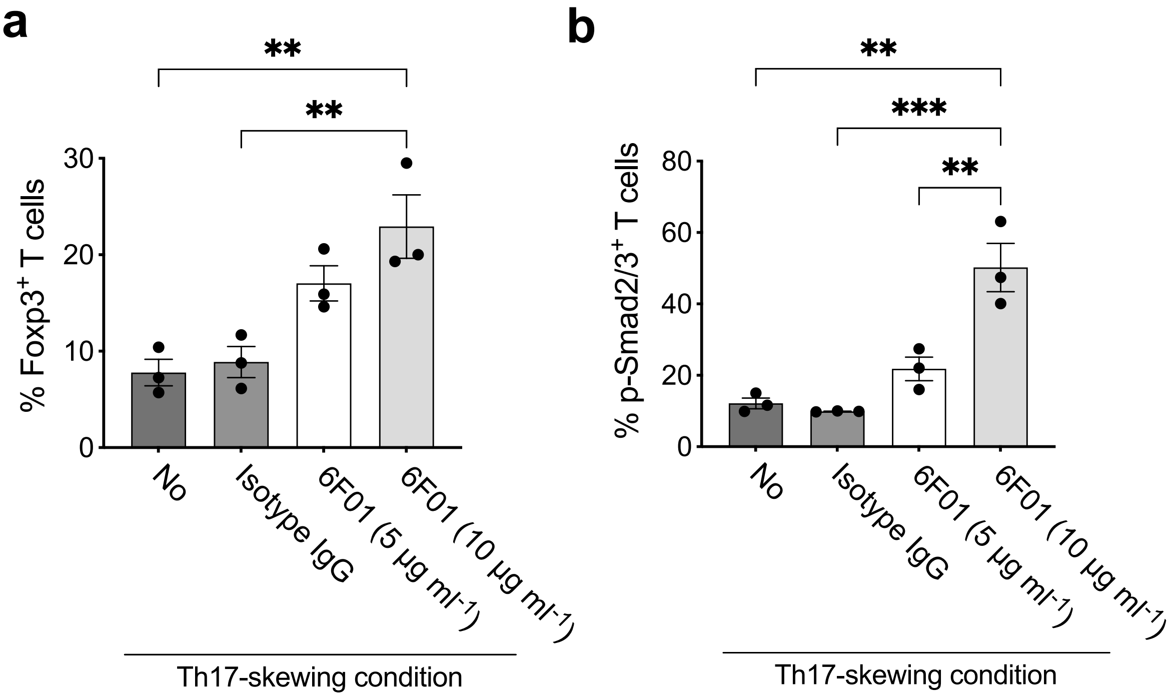


**Supplementary Fig. 14: 6F01 treatment increases Foxp3$^{+}$ and p-Smad2/3$^{+}$ T cell population in Th17 cells. a** The level of Foxp3$^{+}$ T cells by 6F01 stimulation in a dose-dependent manner during Th17 cell differentiation (n=3). Data are expressed as mean ± S.E.M (**P = 0.0063 No vs 6F01, **P = 0.0092 Isotype IgG vs 6F01). **b** The level of Smad2/3 phosphorylation in mouse Th17 cells stimulated with the different concentrations of 6F01 (n=3). Data are expressed as mean ± S.E.M (**P = 0.0011 No vs 6F01, **P = 0.0051 6F01 vs 6F01, ***P = 0.0008). Statistical significance was determined by two-way ANOVA with Tukey's multiple comparisons test (**a, b**). Source data are provided as a Source Data file.

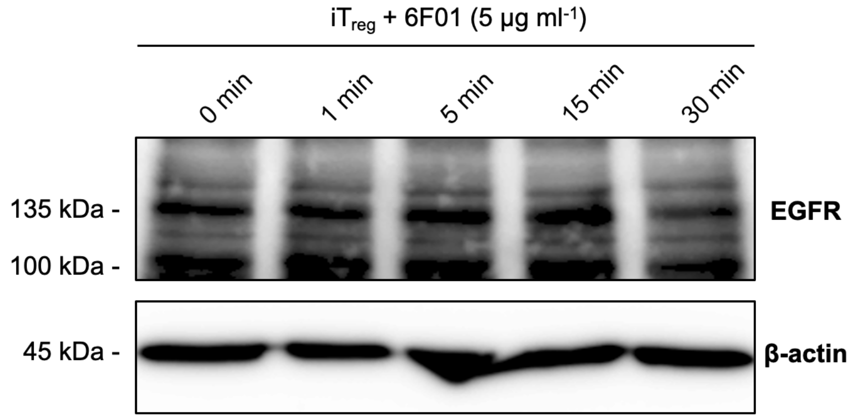


**Supplementary Fig. 15: The short kinetics of EGFR degradation by 6F01 treatment in $iT_{reg}$ cells.** Short-term kinetics analysis of EGFR expression in the differentiated $iT_{reg}$ cells with 6F01 treatment. 3 days-differentiated $iT_{reg}$ cells were incubated in 6F01 (5 μg $ml^{-1}$) coated wells for the indicated times and were lysed with RIPA buffer. Cell lysates were quantified with a BCA protein assay, and the level of EGFR and β-actin was examined by western blot. The experiment was repeated three times independently with similar results. Source data is provided at the end of Supplementary Information.

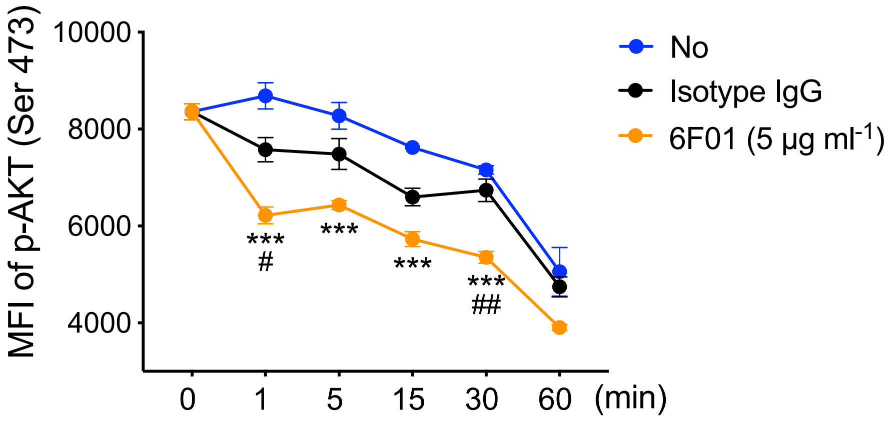


**Supplementary Fig. 16: 6F01 treatment rapidly decreases the phosphorylation of AKT in $iT_{reg}$ cells.** Short-term kinetics analysis of p-AKT (Ser 473) in the differentiated $iT_{reg}$ cells with 6F01 treatment (n=3). 3 days-differentiated $iT_{reg}$ cells were incubated in isotype IgG or 6F01 (5 µg ml$^{-1}$) coated wells for the indicated times and were fixed and permeabilized. Fixed cells were stained by p-AKT antibody and were analyzed by FACS. Data are expressed as mean ± S.E.M (1 min #P = 0.0101, ***P < 0.0001; 5 min ***P = 0.0001; 15 min ***P < 0.0001; 30 min ##P = 0.0079, ***P = 0.0002). * symbol shows versus No and # symbol shows versus Isotype IgG. Statistical significance was determined by two-way ANOVA with Tukey's multiple comparisons test. Source data are provided as a Source Data file.

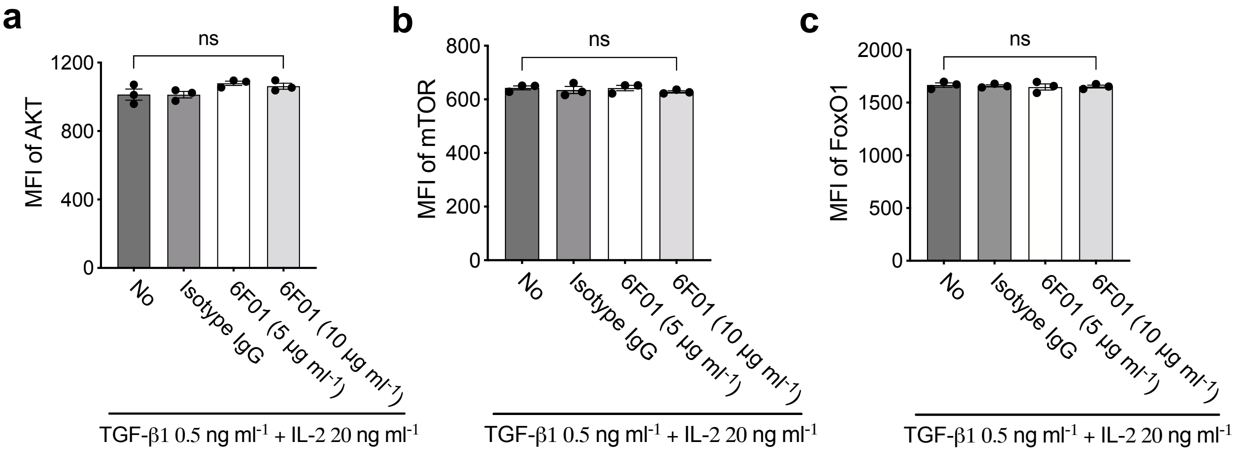


**Supplementary Fig. 17: The protein level of the non-phosphorylated form of AKT, mTOR, or FoxO1 is not affected by the 6F01 treatment. a-c** Mouse CD4$^+$ naïve T cells were differentiated in isotype IgG, or 6F01 (5 or 10 μg ml$^{-1}$) coated well under sub-optimal iT$_{reg}$-skewing condition. After 2 days of differentiation, the cells were harvested and fixed, and permeabilized. The fixed cells were stained with anti-AKT antibody, anti-mTOR antibody, and anti-FoxO1 antibody and were analyzed by FACS (n=3 for each group). Data are displayed as the mean ± S.E.M. ns, not significant. Statistical significance was determined by one-way ANOVA with Tukey's multiple comparisons test (**a-c**). Source data are provided as a Source Data file.

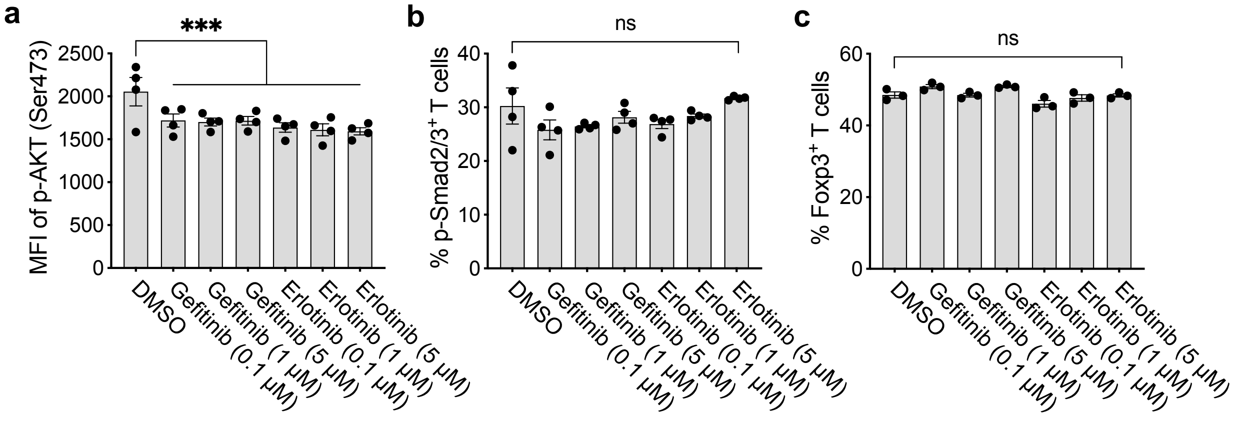


**Supplementary Fig. 18: EGFR inhibitors reduce the phosphorylation of AKT, but do not attenuate the population of p-Smad2/3$^+$ or Foxp3$^+$ T cells. a-c** Mouse CD4$^+$ naïve T cells were differentiated under sub-optimal iT$_{reg}$-skewing conditions. After 24 hours, EGFR-specific inhibitors (Gefitinib and Erlotinib) were treated in a dose-dependent manner. The cells were incubated for additional 24 hours, harvested, fixed, and permeabilized. The fixed cells were stained with an anti-phospho-AKT (Ser473) antibody, anti-phospho-Smad2/3 antibody, and anti-Foxp3 antibody and were analyzed by FACS (n=4 for each group). Data are expressed as mean ± S.E.M (***P < 0.001). Statistical significance was determined by two-way ANOVA with Tukey's multiple comparisons tests (**a-c**). Source data are provided as a Source Data file.

## Supplementary Tables

## Supplementary Table 1. Antibodies used in flow cytometry experiments.

| Marker | Clone | Fluorescence | Company | Dilution |
|---|---|---|---|---|
| CD3 | 17A2 | APC-cy7 | BioLegend | 1:250 |
| CD4 | RM4-4<br>RM4-5 | Brilliant Violet 450<br>FITC/APC | BioLegend<br>Thermo Fisher | 1:250 |
| CD8a | 53-6.7 | FITC | Thermo Fisher | 1:250 |
| CD19 | eBio1D3(1D3) | FITC | Thermo Fisher | 1:250 |
| Foxp3 | FJK-16s | FITC/PE/APC | Thermo Fisher | 1:250 |
| IFNγ | XMG1.2 | FITC/PE/APC | Thermo Fisher | 1:250 |
| IL-4 | 11B11 | PE | Thermo Fisher | 1:250 |
| IL-17A | eBio17B7 | PE | Thermo Fisher | 1:250 |
| IL-10 | JES5-16E3 | PE/APC | Thermo Fisher | 1:200 |
| CD25 | PC61.5 | FITC/PE | Thermo Fisher | 1:250 |
| PD-1 | J43 | PE | Thermo Fisher | 1:250 |
| GITR | DTA-1 | PE | Thermo Fisher | 1:250 |
| TIGIT | GIGD7 | PE | Thermo Fisher | 1:250 |
| CTLA4 | UC10-4B9 | PE | Thermo Fisher | 1:250 |
| CD62L | MEL-14 | FITC | Thermo Fisher | 1:250 |
| CD44 | IM7 | PE/PE-cy7 | Thermo Fisher | 1:250 |
| CD45.1 | A20 | APC-cy7 | BD Biosciences | 1:250 |
| CD45.2 | 104 | FITC | Thermo Fisher | 1:250 |
| Lrig1 | polyclonal | AF488/PE | R&D Systems | 1:100 |
| LRIG1 | 789211 | AF488/PE | R&D Systems | 1:100 |

| | | | | |
|---|---|---|---|---|
| Smad2 (pS465/pS467)/Smad3 (pS423/pS425) | 072-670 | PE | BD Biosciences | 1:100 |
| AKT | C67E7 | unconjugated | Cell Signaling Technology | 1:250 |
| p-AKT1 (Ser473) | SDRNR | APC | Thermo Fisher | 1:250 |
| mTOR | 7C10 | unconjugated | Cell Signaling Technology | 1:400 |
| p-mTOR (Ser2448) | MRRBY | PE-cy7 | Thermo Fisher | 1:250 |
| FoxO1 | C29H4 | unconjugated | Cell Signaling Technology | 1:200 |
| p-FoxO1 (Ser256) | E1F7T | unconjugated | Cell Signaling Technology | 1:250 |

## Supplementary Source Data

### Supplementary Fig. 2c

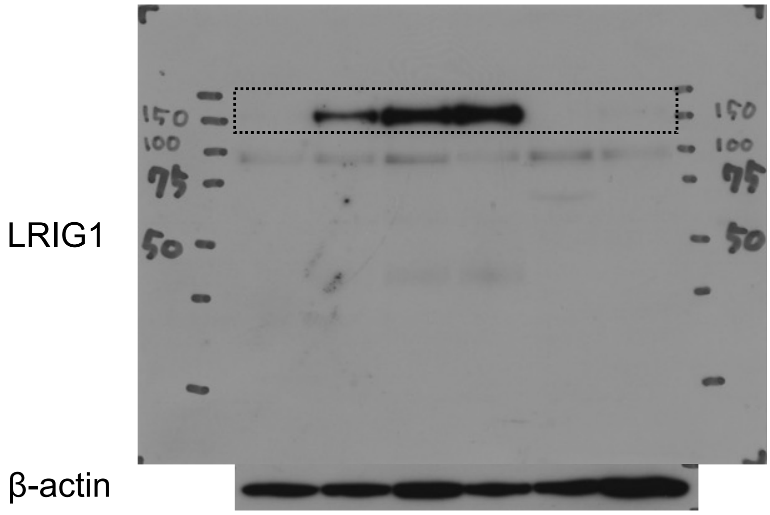


### Supplementary Fig. 15

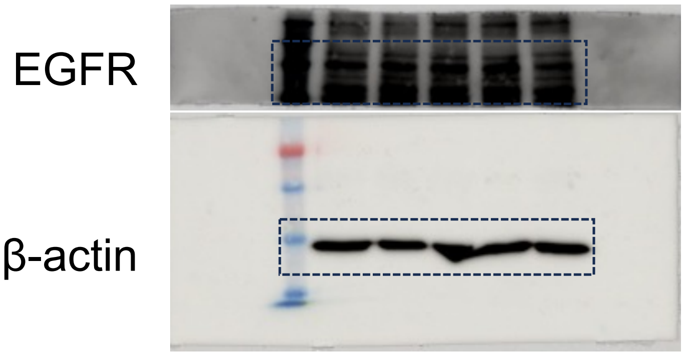

Supplement: Supplementary file 1 — Supplementary Information [file 41467_2023_40986_MOESM1_ESM.pdf]
